# Tissue-resident NK cells differ in their expression profile of the nutrient transporters Glut1, CD98 and CD71

**DOI:** 10.1371/journal.pone.0201170

**Published:** 2018-07-20

**Authors:** Wilhelm Salzberger, Gloria Martrus, Kai Bachmann, Hanna Goebels, Leonard Heß, Martina Koch, Annika Langeneckert, Sebastian Lunemann, Karl J. Oldhafer, Caroline Pfeifer, Tobias Poch, Laura Richert, Christoph Schramm, Ramez Wahib, Madeleine J. Bunders, Marcus Altfeld

**Affiliations:** 1 Department of Viral Immunology, Heinrich Pette Institute, Leibniz Institute for Experimental Virology, Hamburg, Germany; 2 Department of General, Visceral and Thoracic Surgery, University Medical Center Hamburg, Hamburg, Germany; 3 Department of Hepatobiliary and Transplant Surgery, University Medical Center Hamburg, Hamburg, Germany; 4 Department of General & Abdominal Surgery, Asklepios Hospital Barmbek, Semmelweis University of Medicine, Hamburg, Germany; 5 First Department of Medicine, University Medical Center Hamburg-Eppendorf, Hamburg, Germany; 6 INSERM U1219, INRIA SISTM, Bordeaux University, Bordeaux, France; 7 Martin Zeitz Centre for Rare Diseases, University Medical Center Hamburg, Hamburg, Germany; Science and Technology Facilities Council, UNITED KINGDOM

## Abstract

Metabolism is a critical basis for immune cell functionality. It was recently shown that NK cell subsets from peripheral blood modulate their expression of nutrient receptors following cytokine stimulation, demonstrating that NK cells can adjust to changes in metabolic requirements. As nutrient availability in blood and tissues can significantly differ, we examined NK cells isolated from paired blood-liver and blood-spleen samples and compared expression of the nutrient transporters Glut1, CD98 and CD71. CD56^bright^ tissue-resident (CXCR6^+^) NK cells derived from livers and spleens expressed lower levels of Glut1 but higher levels of the amino acid transporter CD98 following stimulation than CD56^bright^ NK cells from peripheral blood. In line with that, CD56^dim^ NK cells, which constitute the main NK cell population in the peripheral blood, expressed higher levels of Glut1 and lower levels of CD98 and CD71 compared to liver CD56^bright^ NK cells. Our results show that NK cells from peripheral blood differ from liver- and spleen-resident NK cells in the expression profile of nutrient transporters, consistent with a cell-adaptation to the different nutritional environment in these compartments.

## Introduction

Natural Killer (NK) cells were first described in 1975 as immune cells able to spontaneously kill tumor cells [1]. Since this first description, it has been shown that NK cells have a key role in several immune-regulation mechanisms, including the ability to recruit other immune cells via cytokine secretion and to kill virus-infected and malignantly transformed cells [2,3]. NK cells furthermore play an important role in reproduction and autoimmunity [4,5]. Classically, NK cells are defined by a lack of CD3, CD14 and CD19 expression combined with expression of CD56 and/or CD16. NK cells are further divided into two main populations, CD56^bright^ CD16^neg^ NK cells and CD56^dim^ CD16^+^ NK cells [6]. In peripheral blood, CD56^bright^ NK cells constitute 10–20% of the NK cell population, and have been suggested to be the precursors of CD56^dim^ NK cells [6]. Upon stimulation, CD56^bright^ NK cells produce high amounts of cytokines and chemokines, which recruit other immune cells and modulate their function [5,7]. In contrast, CD56^dim^ NK cells represent the major NK cell population in peripheral blood, constituting 80–90% of circulating NK cells. CD56^dim^ NK cells are capable of killing target cells via the secretion of perforin and granzymes, and can also produce cytokines such as IFN-γ upon activation, although to a lesser extent than CD56^bright^ NK cells [4,5]. A vast majority of studies on human NK cells have been performed using NK cells derived from peripheral blood. Although convenient to gather functional and phenotypical data, this approach has some limitations, as the major part of the NK cell compartment in the human body resides in tissues [7,8]. A specific population of interest are tissue-resident NK cells, which can reside in tissue for years and execute local tasks. Studies of tissue-resident NK cells from human livers [7–10], lungs [10,11], lymph nodes [12,13], uterus [14], kidneys [15] and spleens [13,16] have elucidated how tissue-resident NK cells are distinct from NK cells circulating in the peripheral blood, showing crucial differences in phenotypical and functional characteristics.

Tissue-resident NK cells are usually defined by the expression of tissue-residency markers, including CD49a, CD69 or CXCR6 [9,17]. Tissue NK cells are therefore divided into two populations, those expressing tissue-residency markers (tissue-resident or TR NK cells) and those NK cells derived from tissues lacking expression of tissue-residency markers (tissue-derived or TD NK cells). Parabiosis studies in mice suggest that the lack of tissue-residency molecules allows NK cells to egress from tissues and re-enter the circulation [18]. Nonetheless, as the microenvironment differs between tissue and blood, these tissue-derived NK cells may acquire some characteristics of tissue-resident NK cells and differ from NK cells observed in peripheral blood, representing a population of transiently tissue-resident NK cells [19]. Tissue-resident NK cells, depending on their specific tissue location, face different nutritional conditions and metabolic requirements than NK cells in peripheral blood. NK cells in peripheral blood usually rely on an abundance of glucose and oxygen, as blood glucose and oxygen levels are tightly regulated in healthy individuals. In contrast, glucose and oxygen levels in tissues need to be constantly replenished via the bloodstream, a process that can be affected by blood circulation, inflammation or tumor infiltration, resulting in local differences of nutrient levels [20,21]. Many tumor cells possess the ability to significantly increase their glucose uptake, thus decreasing glucose availability for immune cells in their surroundings as a mechanism of immune evasion [22]. Similarly, certain viruses such as hepatitis B and C virus can modulate the uptake of glucose into infected cells, which also affects the nutritional microenvironment [23,24]. It still remains unknown how tissue-resident NK cells adjust to these changes in available nutrients to fulfill their functional requirements.

In a resting state, lymphocytes rely on an oxygen-dependent process in the mitochondria, oxidative phosphorylation (OxPhos), in order to efficiently convert one molecule of glucose into 30 molecules of adenosine triphosphate (ATP) [25–28]. When activated, the metabolism of lymphocytes shifts towards glycolysis, even in the presence of oxygen, a process called aerobic glycolysis. During glycolysis, glucose is metabolized to pyruvate and then lactate, which is finally secreted from the cell. Apart from generating ATP, aerobic glycolysis provides precursor molecules essential for the generation of nucleotides, amino acids and lipids [29–31]. However, aerobic glycolysis is less efficient in providing cellular energy, as it only generates two molecules of ATP from each molecule of glucose compared to the 30 molecules of ATP produced per molecule of glucose during OXPHOS [28,29,31]. Inhibition of glycolysis in murine NK cells was shown to prevent proliferation, production of cytotoxic proteins and affected NK cell adhesion [32]. This suggests that tissue-resident NK cells might face the challenge of having high energy requirements during pathological stages while at the same time the availability of glucose can be limited, which might severely inhibit the NK cells functionality.

To measure the metabolic status of lymphocytes, several markers are of interest. Firstly, the glucose uptake from the surrounding microenvironment is controlled in lymphocytes by the glucose transporter 1 (Glut1). Expression of Glut1 was shown to be a critical factor for rapid proliferation and cytokine production in T effector cells [33,34]. A recent study demonstrated that cytokine stimulation of murine splenic and human peripheral blood NK cells upregulated the cell surface expression of Glut1 and increased the rate of glycolysis, oxidative phosphorylation and IFN-γ production [26,35]. However, as most effector functions of NK cells take place in tissues, studies of tissue-derived NK cells are required to understand metabolic pathways impacting NK cell function in tissues. Next to glucose, amino acids can be used for energy generation. Furthermore amino acids are critical building blocks for protein syntheses. CD98 is a transmembrane protein forming a neutral amino acid transport channel when linked with other L-type amino acid transport proteins. Its expression is detected on all cells except from platelets and is upregulated on activated and proliferating lymphocytes [36–39], however the distribution on tissue derived-NK cells is not known. In addition to nutrients for energy production, other nutrients, such as iron, are critical for proliferation and function of lymphocytes. For example intracellular iron is important both in DNA replication but also the expression of costimulatory molecules on the lymphocyte cell surface impacting activation of lymphocytes [40–42]. Due to its toxicity at higher concentrations, cellular iron content is tightly regulated [40]. Cell surface transferrin receptor protein 1 (TfR1) or CD71 is required for uptake of diferric transferrin via receptor-mediated endocytosis [40,41]. CD71 expression can be further used as a marker for activation or proliferation of T cells [41,43] and was recently reported to be upregulated on cytokine-stimulated NK cells derived from peripheral blood in parallel with an upregulation of CD98 and Glut1 [35]. In this study, we examine whether NK cells from spleen and liver tissues differ from peripheral blood NK cells in their expression profile of the nutrient transporters Glut1, CD98 and CD71 in steady state, and in their ability to upregulate these nutrient transporters in response to cytokine stimulation.

## Methods

### Patients and ethics

Matched liver and paired blood samples (n = 12) were obtained from individuals undergoing liver transplantation in the Department of Hepatobiliary and Transplant surgery at the University Medical Center Hamburg-Eppendorf or individuals undergoing liver resection in the process of abdominal tumor excision surgery at the Asklepios Hospital, Barmbek (Table 1). Matched spleen and blood samples (n = 11) were obtained from individuals undergoing abdominal excision surgery at the Department of General, Visceral and Thoracic surgery at the University Medical Center Hamburg-Eppendorf (Table 2). All samples used in this study were obtained from patients undergoing surgery for medical reasons. All study participants provided informed written consent according to the guidelines by the Institutional Review Board of the medical faculty at the University of Hamburg. This study received ethics approval under the ethic proposals PV3548 (spleen samples) and PV4898 (liver samples) by the Ärztekammer Hamburg.

**Table 1:** Relevant clinical data of the liver cohort.

Internal Code	Sample Type	Gender	Age	Diagnosis
L1	Liver+Blood	M	73	Malignant neoplasia of the liver after rectal carcinoma
L2	Liver+Blood	M	63	Hepatocellular carcinoma
L3	Liver+Blood	M	64	Retransplant after autoimmune hepatitis / primary sclerosing cholangitis
L4	Liver+Blood	M	57	Hepatocellular carcinoma (state after hepatitis E infection)
L5	Liver+Blood	M	59	Hepatocellular carcinoma
L6	Liver+Blood	M	64	Hepatocellular carcinoma
L7	Liver+Blood	M	43	Retransplant after hydropic decompensation of transplant after Morbus Wilson
L8	Liver+Blood	M	54	Decompensated livercirrhosis after hepatitis B and C infection
L9	Liver+Blood	M	58	Hepatocellular carcinoma after hepatitis C infection
L10	Liver+Blood	F	46	Primary biliary cholangitis
L11	Liver+Blood	F	51	Polycystic liver disease
L12	Liver+Blood	F	33	Drug induced acute liver failure
Median (IQR)			57 (47–63)	

**Table 2:** Relevant clinical data of the spleen cohort.

Internal Code	Sample Type	Gender	Age	Diagnosis
S1	Spleen+Blood	M	52	Gastrointestinal stromal tumor
S2	Spleen+Blood	F	68	Intraductal papillary mucinous neoplasia of the pancreas
S3	Spleen+Blood	M	79	Non-Hodgkin B-Cell Lymphoma
S4	Spleen+Blood	M	80	Diffuse gastric cancer
S5	Spleen+Blood	F	74	Pancreatic adenocarcinoma
S6	Spleen+Blood	F	75	Gastroesophageal junctional adenocarcinoma
S7	Spleen+Blood	M	51	Gastrointestinal stromal tumor
S8	Spleen+Blood	F	31	Chronic immune thrombocytopenic purpura
S9	Spleen+Blood	F	35	Steroid-refractory immune thrombocytopenic purpura
S10	Spleen+Blood	F	73	Immune thrombocytopenic purpura with splenomegaly with Non-Hodgkin B Lymphoma
S11	Spleen+Blood	F	44	Dilated lymphatic vessels
Median (IQR)			68 (44–75)	

### Isolation of mononuclear cells from blood and tissue

Peripheral blood mononuclear cells (PBMCs) were isolated from full blood via density centrifugation using Ficoll/Percoll. After counting, cells were frozen in 90%FBS/10% DMSO and stored in liquid nitrogen for later use. Mononuclear cells from spleen tissue (SMCs) were isolated by cutting the fresh tissue into pieces of approximately 0.5x0.5x0.5cm. Spleen tissue pieces were then manually pushed through a series of filters (5003bcm/300μm/100μm/70μm/40μm, Greiner Bio-One GmbH) with a plunger while irrigating the samples with Hanks solution when needed. After washing, the cells underwent erythrocyte lysis using ACK lysis buffer (Biozym) for 3 minutes and were subsequently washed twice with Hanks solution. Cells were then counted with a TC20 automated cell counter (Biorad) and their viability was assessed with trypan blue staining before cryopreservation for later use. Mononuclear cells from liver tissue (LMCs) were isolated by manually cutting tissues into pieces of approximately 0.5x0.5x0.5cm. Liver tissue pieces were then mechanically dissociated with a gentleMACS Octo Dissociator (Milteny). After the mechanical dissociation, cells were manually pushed through a series of filters (500μm/300μm/100μm/70μm/40μm). After washing, LMCs were isolated via a series of density centrifugation steps with Optiprep density gradient medium (Sigma-Aldrich), counted and frozen for later use.

### Culturing and stimulation

All cells were cultured in RMPI 1640 (Life Technologies GmbH), supplemented with 10% heat inactivated FBS Superior (Biochrom AG). Cells were stimulated with 5ng/mL of recombinant human IL-12 and 2.5ng/mL of recombinant IL-15 (both from Pepro Tech EC GmbH).

### Flow stainings

Surface antibody stainings were performed in PBS supplemented with 2% heat inactivated FBS for 20 minutes at room temperature. Glut1.- receptor dinding protein (RBD)-eGFP labeling was done in RPMI1640 + 10%FBS + 5% NaN_3_ +0.5mM EDTA for 30 minutes at 37°C. The eBioscience Foxp3/Transcription Factor Staining Buffer Set (product protocol B) was used for intracellular staining. The monoclonal mouse anti-human antibodies anti-CD16-BUV395 (clone 3G8, BD Horizon^TM^), anti-CD56-BUV737 (clone NCAM16.2, BD Horizon^TM^), anti-CD71-BV711 (clone M-A712, BD Horizon^TM^), anti-CD57-BV605 (clone NK-1, BD Horizon^TM^), anti-CD186 (CXCR6)-PE/Cy7 (clone K041E5, Biolegend), anti-CD127-PE-CF594 (clone HIL-7R-M21, BD Horizon^TM^), anti-CD45-BV785 (clone HI30, Biolegend), anti-CD3-AlexaFluor700 (clone UCHT1, Biolegend), anti-CD14-BV510 (clone M5E2, Biolegend), anti-CD19-BV510 (clone HIB19, Biolegend), as well as recombinant human anti-CD98-APC-Vio770 (clone REA387, Milteny Biotec GmbH), Zombie Aqua™(Biolegend) and anti-human GLUT1 (SLC2A1)-eGFP RBD were used for staining. NK cells were defined as CD3 ^neg^, CD14 ^neg^, CD19 ^neg^, CD45+, and were divided into CD56^bright^ CD16 ^neg^ NK cells (referred to as CD56^bright^ NK cells) and CD56^dim^ CD16+ NK cells (referred to as CD56^dim^ NK cells).

### Analysis

Samples were acquired on a LSR Fortessa (BD Biosciences) and results were analyzed using Flowjo software version 10. Percentages of subpopulations and median fluorescence intensity (MdFI) values of subpopulations from paired samples were compared using Wilcoxon matched-pairs signed rank tests using Graphpad Prism version 6. Test multiplicity was controlled for by a false discovery rate (FDR) method accounting for dependency among statistical tests, using SAS software, version 9.3 (SAS Institute, Cary, North Caroline, USA)[44]. A p-values reported in this manuscript are FDR-adjusted and considered statistically significant if p < 0.05. Cytobank services were used for viSNE analysis.

## Results

### Phenotypic characteristics of NK cells from liver tissue, spleen tissue and peripheral blood

**Fig 1:**
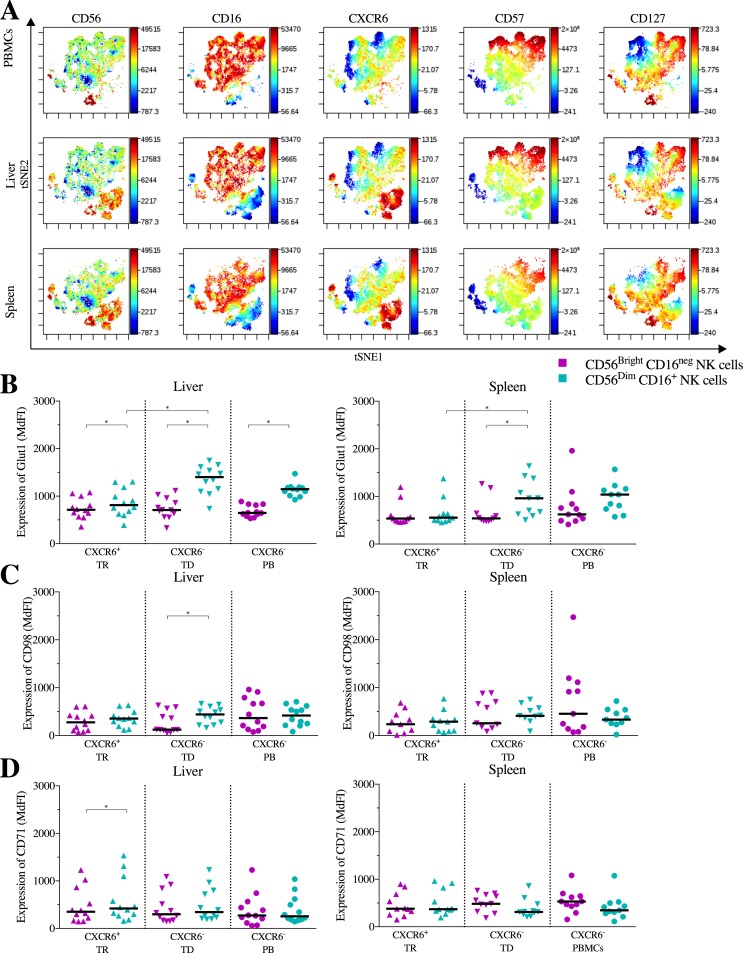
NK cell phenotype and baseline nutrient receptor expression: Samples were compared using Wilcoxon matched-pairs signed rank tests and multiplicity was controlled for by FDR testing. Bars indicate the median, significance was defined as p≤0.05 (*). A. viSNE representation of peripheral blood- (PBMC, top row), liver- (middle row) and spleen (bottom row) derived NK cells and their expression of CD56, CD16, CXCR6, CD57 and CD127. B. Expression (Median fluorescence intensity, MdFI) of Glut1 on CD56^bright^CD16^-^ (purple) and CD56^dim^CD16^+^ (teal) tissue-resident (TR), tissue-derived (TD) and peripheral blood (PB) NK cells from paired liver-blood (left diagram, n = 12) and spleen-blood (right diagram, n = 11) samples. C. Expression (MdFI) of CD98 on CD56^bright^CD16^-^ (purple) and CD56^dim^CD16^+^ (teal) tissue-resident (TR), tissue-derived (TD) and peripheral blood (PB) NK cells from paired liver-blood (left diagram, n = 12) and spleen-blood (right diagram, n = 11) samples. D. Expression (MdFI) of CD71 on CD56^bright^CD16^-^ (purple) and CD56^dim^CD16^+^ (teal) tissue-resident (TR), tissue-derived (TD) and peripheral blood (PB) NK cells from paired liver-blood (left diagram, n = 12) and spleen-blood (right diagram, n = 11) samples.

NK cells in peripheral blood are predominantly CD56^dim^CD16^+^ NK cells and only a very low percentage of NK cells express tissue-residency markers such as CD49a, CD69 or CXCR6. NK cells from liver and spleen tissues on the other hand have been reported to contain a sizable population of CD56^bright^CD16^neg^ NK cells as well as NK cells expressing tissue residency markers [7,8,13]. To identify tissue-resident and circulating NK cells, we analyzed the distribution of CD56^dim^ and CD56^bright^ NK cells as well as the expression of CXCR6, a marker for tissue residency [45], in peripheral blood, liver tissue and spleen tissue samples. We separated NK cells into two subsets, CD56^bright^ and CD56^dim^ NK cells. CXCR6^+^ NK cells from spleen and liver tissues were classified as tissue-resident (TR) NK cells. CXCR6^neg^ NK cells derived from spleen and liver tissues were classified as tissue-derived (TD) NK cells, CXCR6^neg^ NK cells from the blood were classified as peripheral blood (PB) NK cells. An enrichment of the proportion of CD56^bright^ NK cells in tissue samples compared to peripheral blood samples was observed (PB vs. TR liver: p = 0.02; PB vs. TR spleen: p = 0.02, Table 3). CD56^bright^ NK cells from liver and spleen tissues were predominantly CXCR6^+^ tissue-resident NK cells compared to peripheral blood (blood vs. liver: p = 0.02; blood vs. spleen: p = 0.03; Table 3), while CD56^dim^ NK cells from liver and spleen tissues were predominantly CXCR6^neg^ tissue-derived NK cells, although there were significantly more CD56^dim^ CD16^+^ CXCR6^+^ NK cells present in both liver and spleen tissues compared to peripheral blood (PB vs. TR liver: p = 0.03; PB vs. TR spleen: p = 0.03; Table 3). In peripheral blood, only a small fraction of NK cells expressed CXCR6 (Table 3). No significant differences in the expression of the differentiation markers CD57 and CD127 between CD56^bright^ CD16^neg^ from liver, spleen and peripheral blood or CD56^dim^ CD16^+^ NK cells from these compartments were observed (Fig 1A and data not shown). Taken together, these results confirmed previous studies [5,8,46] showing that peripheral blood contains mostly CXCR6^neg^ CD56^dim^ NK cells, while both spleen and liver tissues contain CD56^bright^ and CD56^dim^ NK cells, including a significant number of NK cells expressing the tissue-residency marker CXCR6.

**Table 3:** Expression of CXCR6 in NK cells in tissues.

		%CD56^bright^ NK cells	%CXCR6^+^ among CD56^bright^ NK cells	%CXCR6^+^ among CD56^dim^ NK cells
**Liver (n = 12)**	**Tissue**	37.3% (30.0–45.2%)	76.8% (52.0–86.5%)	22.8% (4.8–36.3%)
	**Blood**	4.9% (3.4–6.8%)	8.2% (2.4–24.0%)	1.6% (0.9–2.8%)
**Spleen (n = 11)**	**Tissue**	29.1% (18.2–42.7%)	67.3 (35.8–74.3%)	8.8% (5.0–10.2%)
	**Blood**	3.7% (1.4–5.2%)	6.6% (1.6–17.5%)	0.6% (0.4–1.1%)

Median and interquartile range (IQR) of %CD56^bright^ NK cells, %CXCR6^+^ among CD56^bright^ NK cells and %CXCR6^+^ among CD56^dim^ NK cells in tissue and blood of liver and spleen tissue donors

### Expression of nutrient transporters on NK cells from liver, spleen and peripheral blood

The expression of transporters for nutrients can vary between different NK cell subsets [35]. In peripheral blood, nutrients are usually readily available, while nutrient levels in tissues depend on replenishment via the blood and *in situ* neogenesis, and can vary depending on blood flow and local metabolic conditions [20]. We examined the expression of Glut1, an important transporter for glucose into cells, CD98, a transporter for amino acids, and CD71, a transporter for transferrin, on NK cells derived from peripheral blood and patient-matched liver and spleen tissues.

In general, a lower expression of Glut1 on CXCR6^+^ compared to CXCR6^neg^ CD56^dim^ NK cells isolated from liver and spleen tissues was observed (liver: p = 0.02; spleen: p = 0.05; Fig 1B). Glut1 was also expressed at lower levels on tissue-resident CXCR6^+^ CD56^dim^ NK cells from tissues compared to peripheral blood CD56^dim^ NK cells, although these differences did not reach statistical significance (liver: p = 0.1; spleen: p = 0.2). In contrast, no differences in Glut1 expression were observed between CD56^bright^ NK cells derived from blood, liver and spleen, neither in the CXCR6^+^ nor CXCR6^neg^ CD56^bright^ NK cell populations. However, when assessing CD56^bright^ and CD56^dim^ NK cells from the same tissue compartments, a significantly lower expression of Glut1 on CD56^bright^ NK cells compared to CD56^dim^ NK cells was observed for all NK cell subsets in livers (CXCR6^+^: p = 0.02; CXCR6^neg^: p = 0.02; PB: p = 0.01; Fig 1B) and in spleens for CXCR6^neg^ CD56^bright^ NK cells (p = 0.03; Fig 1B). While CD98 expression differed slightly between different NK cell subsets, these differences represented only minor shifts in the MdFI of CD98 (Fig 1C). Furthermore, no significant differences in the expression of CD71 were observed between CXCR6^+^ or CXCR6^neg^ NK cells derived from tissues and peripheral blood NK cells at baseline, apart from a slightly higher expression of CD71 on CXCR6^+^ CD56^dim^ compared to CXCR6^+^ CD56^bright^ NK cells derived from livers (p = 0.02; Fig 1D). In conclusion, and in contrast to prior reports [35], we consistently observed a higher expression of Glut1 at baseline, without any stimulation, on CD56^dim^ compared to CD56^bright^ NK cells in most investigated tissue compartments. Furthermore, tissue-resident CXCR6^+^ CD56^dim^ NK cells expressed significantly lower levels of Glut1 compared to tissue-derived CXCR6^neg^ CD56^dim^ NK cells.

### Effect of cytokine stimulation on NK cell phenotype and Glut1 expression

**Fig 2:**
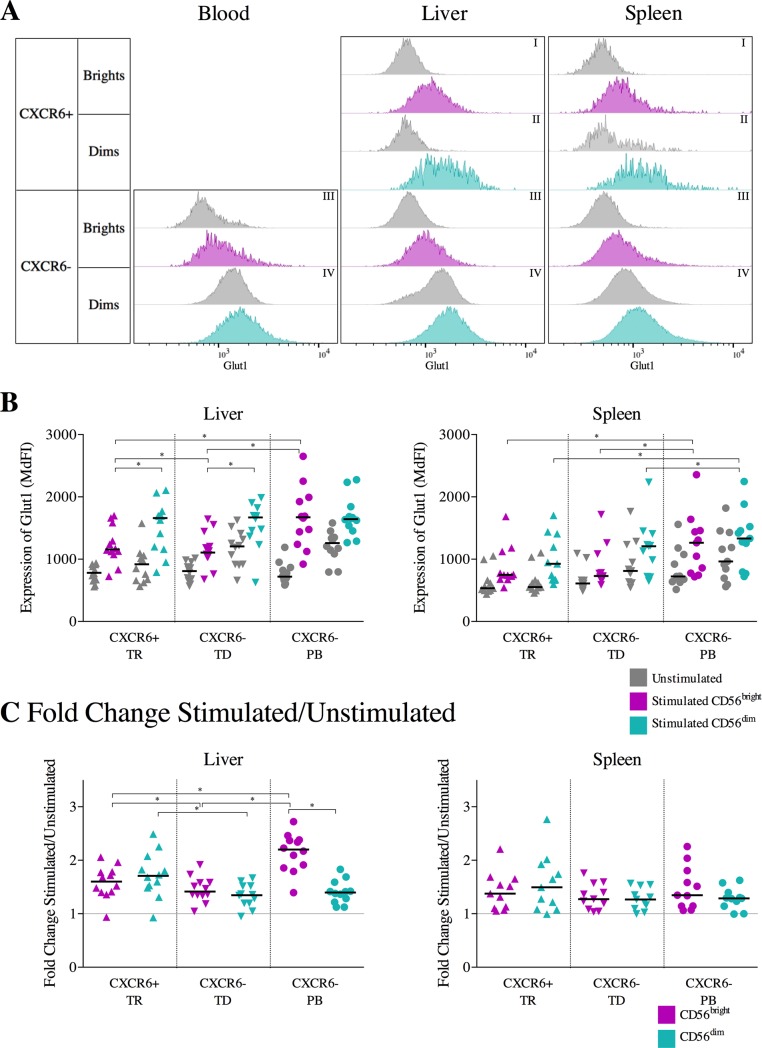
Effects of cytokine stimulation on Glut1 expression: Samples were compared using Wilcoxon matched-pairs signed rank tests and multiplicity was controlled for by FDR testing. Bars indicate the median, significance was defined as p≤0.05 (*). A. Representative histograms of Glut1 expression on unstimulated and stimulated CD56^bright^ CXCR6^+^ (I, grey: unstimulated; purple: stimulated), CD56^dim^ CXCR6^+^ (II, grey: unstimulated; teal: stimulated), CD56^bright^ CXCR6^-^ (III, grey: unstimulated; purple: stimulated) and CD56^dim^ CXCR6^-^ (IV, grey: unstimulated; teal: stimulated) NK cells from blood (left), liver (middle) and spleen (right) samples. B. Expression (MdFI) of Glut1 on unstimulated (grey) and CD56^bright^CD16^-^ (purple) and CD56^dim^CD16^+^ (teal) tissue-resident (TR), tissue-derived (TD) and peripheral blood (PB) NK cells from paired liver-blood (left diagram, n = 12) and spleen-blood (right diagram, n = 11) samples. C. Differences (fold change) in Glut1 expression (MdFI) between stimulated and unstimulated CD56^bright^CD16^-^ (purple) and CD56^dim^CD16^+^ (teal) tissue-resident (TR), tissue-derived (TD) and peripheral blood (PB) NK cells from paired liver-blood (left diagram, n = 12) and spleen-blood (right diagram, n = 11) samples.

We next determined the ability of peripheral blood and tissue NK cell subsets to regulate the expression of nutrient transporters, as it was previously reported that stimulation of NK cells induces shifts in their expression profiles [35]. We first studied the effect of cell culture and cytokine stimulation on NK cell subset distributions and phenotypes. Overnight incubation, including stimulation with low amounts of cytokines (5ng/mL IL-12 and 2.5ng/mL IL-15), induced no significant changes in the proportion of CD56^bright^ and CD56^dim^ NK cells derived from all tissue compartments (Fig 2B and S2 Table). In the overnight cell cultures lacking cytokines, a significant decrease of CXCR6^+^ CD56^bright^ NK cells was observed (liver: p = 0.01; spleen: p = 0.04; S2 Table), while the percentage of CXCR6^+^ CD56^bright^ and CD56^dim^ NK cells derived from peripheral blood increased (p = 0.01 for both subsets; S2 Table). Incubation in cytokine-free medium did not induce any significant changes in Glut1-expression on any NK cell population (Part A in S1 Fig). Taken together, these data (Fig 1B and Part A in S2 Fig) show that, in the absence of any stimulation, tissue-resident CD56^dim^ NK cells expressed lower levels of Glut1 than peripheral blood and tissue-derived CD56^dim^ NK cells, while CD56^bright^ NK cells did not differ in their expression of Glut1 irrespective of tissue-residency.

To determine the impact of cytokine stimulation on nutrient transporter expression profiles of NK cells, we next investigated the expression of Glut1 after overnight stimulation with IL-12 and IL-15 (Fig 2A). Cytokine stimulation with 5ng/mL IL-12 and 2.5ng/mL IL-15 over 18 hours led to a significant upregulation of Glut1-expression on all NK cell subsets compared to their unstimulated counterparts (Fig 2B and Part A in S2 Fig). Glut1 expression upon stimulation was higher on CD56^dim^ NK cells compared to CD56^bright^ NK cells in tissues, for example liver-resident CD56^dim^ NK cells compared to liver-resident CD56^bright^ NK cells (p = 0.02; Fig 2B). However, when assessing the fold change as a measure of capacity to upregulate Glut1 after cytokine stimulation, in particular peripheral blood-derived CD56^bright^ NK cells were more effective in upregulating Glut1 after cytokine stimulation compared to CXCR6^+^ or CXCR6^neg^ CD56^bright^ NK cells from liver (p < 0.05 for all comparisons, Fig 2B). Peripheral blood CD56^bright^ NK cells also expressed significantly higher Glut1 levels after cytokine stimulation compared to tissue-resident CXCR6^+^ or tissue-derived CXCR6^neg^ CD56^bright^ NK cells from liver (p < 0.05 for all comparisons, Fig 2B). Liver-resident CXCR6^+^ CD56^bright^ NK cells showed a significantly higher upregulation of Glut1 when compared to liver-derived CXCR6^neg^ CD56^bright^ NK cells (p = 0.04), but overall a significantly lower upregulation of Glut1 than peripheral blood CD56^bright^ NK cells (p = 0.03, Fig 2C). Furthermore, even after cytokine stimulation, within the liver, CD56^bright^ NK cells still expressed significantly less Glut1 than CD56^dim^ NK cells (p = 0.02 for both TR and TD; Fig 2B). A similar tendency was observed for NK cells derived from spleens, with CD56^dim^ NK cells expressing higher levels of Glut1 than CD56^bright^ NK cells, irrespectively of the tissue compartment (TR: p = 0.5; TD: p = 0.07; Fig 2B). In addition, CXCR6^+^ and CXCR6^neg^ CD56^dim^ NK cells derived from spleens expressed significantly less Glut1 after cytokine stimulation compared to peripheral blood NK cells (p = 0.02 for both comparisons; Fig 2B). Overall, CD56^dim^ NK cells had a higher expression of Glut1 than CD56^bright^ NK cells, however after stimulation, peripheral blood CD56^bright^ NK cells were most effective in upregulating Glut1 while the large population of CD56^bright^ NK cells from tissues expressed significantly lower amounts of Glut1.

### Effect of cytokine stimulation on expression of CD98 on NK cells from liver, blood and spleen

**Fig 3:**
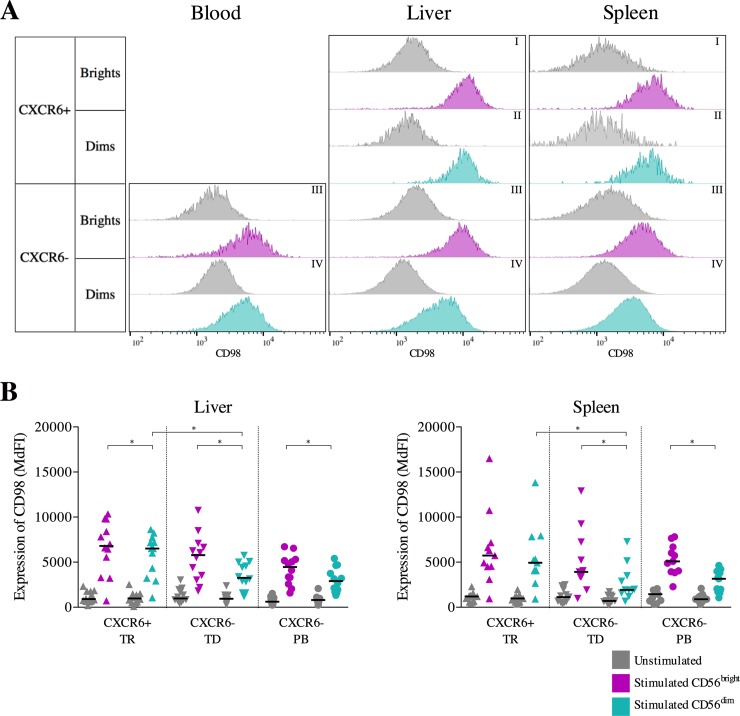
Effects of cytokine stimulation on CD98 expression: Samples were compared using Wilcoxon matched-pairs signed rank tests and multiplicity was controlled for by FDR testing. Bars indicate the median, significance was defined as p≤0.05 (*). A. Representative histograms of CD98 expression on unstimulated and stimulated CD56^bright^ CXCR6^+^ (I, grey: unstimulated; purple: stimulated), CD56^dim^ CXCR6^+^ (II, grey: unstimulated; teal: stimulated), CD56^bright^ CXCR6^-^ (III, grey: unstimulated; purple: stimulated) and CD56^dim^ CXCR6^-^ (IV, grey: unstimulated; teal: stimulated) NK cells from blood (left), liver (middle) and spleen (right) samples. B. Expression (MdFI) of CD98 on unstimulated (grey) and CD56^bright^CD16^-^ (purple) and CD56^dim^CD16^+^ (teal) tissue-resident (TR), tissue-derived (TD) and peripheral blood (PB) NK cells from paired liver-blood (left diagram, n = 12) and spleen-blood (right diagram, n = 11) samples.

We next examined changes in the expression of CD98 on NK cells derived from livers, spleens and peripheral blood stimulated with IL-12 and IL-15 (Fig 3A). Incubation in cytokine-free medium induced a significant but small increase of CD98 expression in the majority of NK cell subsets (Part B in S1 Fig). In contrast, overnight cytokine stimulation led to a significant upregulation of CD98 (Fig 3B and Part B in S2 Fig) expression in all NK cell subsets. Tissue-resident, tissue-derived and peripheral blood CD56^bright^ NK cells had a higher expression of CD98 than tissue-resident, tissue-derived and peripheral-blood CD56^dim^ NK cells from liver (TR: p = 0.04; TD: p = 0.02; PB = 0.02; Fig 3B) and spleen donors (TD: p = 0.02; PB: p = 0.02; Fig 3B), although this did not reach significance for spleen resident NK cells. Furthermore, tissue-resident CXCR6^+^ CD56^dim^ NK cells from liver and spleen had a higher expression of CD98 compared to tissue-derived CXCR6^neg^ (liver: p = 0.02; spleen: p = 0.02; Fig 3B). Taken together, cytokine stimulation led to a robust upregulation of CD98 on NK cells, with highest levels in tissue-resident and tissue-derived CD56^bright^ NK cells in the liver.

### Effect of cytokine stimulation on expression of CD71 on NK cells from liver, blood and spleen

**Fig 4:**
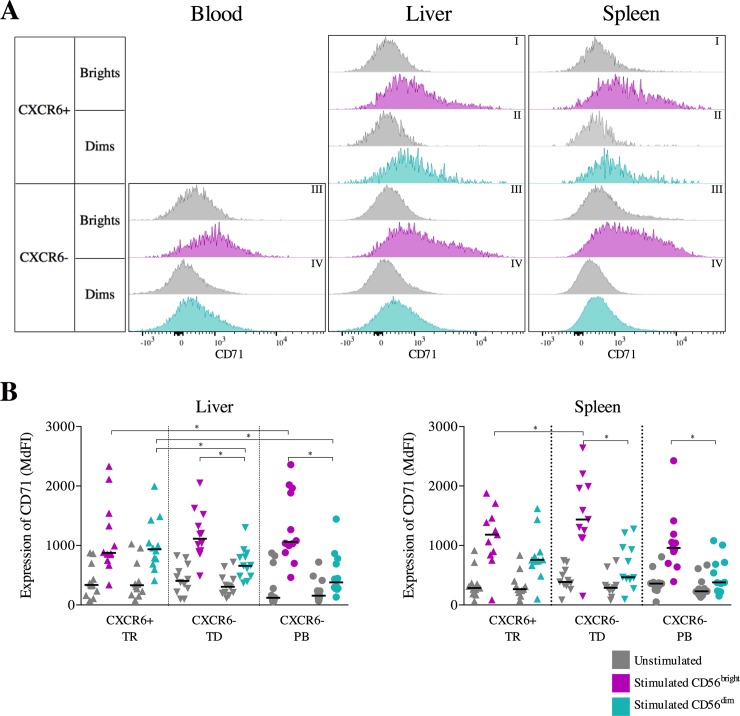
Effects of cytokine stimulation on CD71 expression: Samples were compared using Wilcoxon matched-pairs signed rank tests and multiplicity was controlled for by FDR testing. Bars indicate the median, significance was defined as p≤0.05 (*). A. Representative histograms of CD71 expression on unstimulated and stimulated CD56^bright^ CXCR6^+^ (I, grey: unstimulated; purple: stimulated), CD56^dim^ CXCR6^+^ (II, grey: unstimulated; teal: stimulated), CD56^bright^ CXCR6^-^ (III, grey: unstimulated; purple: stimulated) and CD56^dim^ CXCR6^-^ (IV, grey: unstimulated; teal: stimulated) NK cells from blood (left), liver (middle) and spleen (right) samples. B. Expression (MdFI) of CD71 on unstimulated (grey) and CD56^bright^CD16^-^ (purple) and CD56^dim^CD16^+^ (teal) tissue-resident (TR), tissue-derived (TD) and peripheral blood (PB) NK cells from paired liver-blood (left diagram, n = 12) and spleen-blood (right diagram, n = 11) samples.

Lastly, we assessed the expression of the transferrin transporter CD71 on NK cells from blood, liver and spleen tissue after overnight incubation with and without cytokines (Fig 4A). Incubation in cytokine-free medium induced no significant changes in CD71 expression (Part C in S1 Fig). However, stimulation with cytokines also led to a significant upregulation of CD71 in all NK cell subsets (Fig 4B and Part C in S2 Fig). Overall, CD56^bright^ NK cells had the highest CD71 expression compared to CD56^dim^ NK cell populations, and this was most pronounced amongst spleen-derived CXCR6^neg^ cells (p = 0.02, Fig 4B). A similar trend was observed in liver-derived CXCR6^neg^ NK cell population (p = 0.02, Fig 4B). Thus CD71 expression was higher on tissue-resident CXCR6^+^ CD56^dim^ NK cells compared to tissue-derived CXCR6^neg^ CD56^dim^ NK cells, indicating a difference between tissue-resident and tissue-derived CD56^dim^ NK cells in their capacity for nutrient uptake.

## Discussion

Immune cell metabolism is a critical determinant of the function of tissue-resident immune cells, as nutrient availability in tissues can largely vary depending on the specific tissue characteristics and other factors influencing local nutrient availability, such as viral infections [23,24,47] and malignancies [22,48,49]. Infections and malignancies affect the nutritional microenvironment by changing nutrient supply to tissues, resulting from fibrosis or modulated vascularization [50,51], or by consuming nutrients themselves [48]. When activated, human immune cells, including NK cells, are able to radically increase their nutrient uptake in order to act efficiently and quickly. On the other hand, it was demonstrated that inhibition of glycolysis can decrease NK cell functionality against viral targets [32]. In the last years, important differences in phenotypes and functions of NK cell subsets from different tissue compartments have been elucidated [8,9,12,14,17]. However, no study on differences in the expression of nutrient transporters on NK cells derived from different tissues has been performed to date. In this study, we characterized the expression of the metabolic transporters Glut1, CD98 and CD71 on unstimulated and stimulated NK cell subsets derived from peripheral blood, liver and spleen obtained from individuals undergoing liver transplantation, liver resection in the process of abdominal tumor excision surgery, or spleen resection for medical reasons. We observed significant differences between tissue-resident, tissue-derived and peripheral CD56^dim^ and CD56^bright^ NK cells in regard to their expression of nutrient transporters, both before and after stimulation with cytokines.

To characterize the expression of nutrient transporters on tissue-resident and tissue-derived NK cells, we initially studied NK cells from liver and spleen tissues and compared these samples to paired blood samples without any prior incubation or stimulation. Glut1 is one of the transporters for glucose into cells and expressed on virtually all human cells. Its expression level on T cells has been linked to their functional capacity [52] and Glut1 expression has been demonstrated to differ between NK cell subsets in peripheral blood [35]. When comparing tissue and peripheral blood NK cells without any prior incubation or stimulation, we observed a higher expression of Glut1 on CD56^dim^ NK cells compared to CD56^bright^ NK cells in peripheral blood and the CXCR6^neg^ population of NK cells derived from tissues. Tissue-resident CXCR6^+^ NK cells on the other hand had a low expression of Glut1, irrespective of their expression level of CD56. We next examined the effect of overnight incubation with IL-12 and IL-15 on the expression of Glut1 on NK cells from livers, blood and spleens. Previous studies had shown that peripheral blood NK cells can upregulate Glut1 expression in response to a combination of these cytokines, which went in hand with functional changes, such as IFNγ production [26,35]. While the differences in Glut1 expression between NK cell subsets were modest, our data show that NK cells from peripheral blood either expressed high levels of Glut1 already in the resting state (CD56^dim^ NK cells) or upregulated Glut1 expression to a high degree when stimulated (CD56^bright^ NK cells). On the other hand, NK cells derived from tissues, especially CD56^bright^ NK cells, did not express similar levels of Glut1 after stimulation, suggesting that these tissue-resident NK cells might be less dependent on the ability to upregulate their glucose uptake upon activation and instead use other metabolites. In peripheral blood, glucose levels are usually maintained at a high level [53], enabling peripheral blood NK cells to rely in their metabolism on glucose when needed. NK cells from tissues on the other hand are more likely to encounter situations in which glucose might not be readily available [53,54], resulting in metabolic challenges that could impair NK cell functions. Tissue-resident NK cells therefore might be already adapted to conditions in which glucose is rare by using other nutrients than glucose in order to maintain their anti-viral and anti-tumor activity.

One important alternative source of energy apart from glucose are amino acids. CD98 is a molecule composing a transporter system for amino acids into the cell [55]. Recently, it was reported that CD98 is upregulated along with Glut1 and CD71 on cytokine-stimulated peripheral blood NK cells [35]. Additionally, it was demonstrated that murine splenic NK cells possess the ability to switch from glycolysis towards non-glucose based oxidative phosphorylation when glycolysis was inhibited *in vitro* [36,56]. While no differences in expression levels of CD98 were observed between CD56^dim^ and CD56^bright^ NK cells stained without any prior incubation or stimulation, peripheral blood CD56^bright^ NK cells expressed significantly higher CD98 levels than peripheral blood CD56^dim^ NK cells upon cytokine stimulation, in line with previously published results [35]. We furthermore observed a higher expression of CD98 on stimulated liver- and spleen-resident CXCR6^+^ CD56^dim^ NK cells compared to tissue-derived CXCR6^neg^ CD56^dim^ NK cells. Our results showed that CD56^bright^ NK cells derived from livers and spleens tissues preferentially expressed CD98 post-cytokine stimulation compared CD56^dim^ NK cells. Moreover, we observed that tissue-resident CD56^dim^ NK cells from the liver expressed significantly higher CD98 levels than tissue-derived and peripheral blood CD56^dim^ NK cells, although they expressed less CD98 than CD56^bright^ NK cells from the respective compartments. Taken together, CD56^bright^ tissue-resident NK cells, which expressed lower amounts of Glut1 expressed high amounts of CD98 after stimulation. In contrast, NK cell subsets expressing high Glut1 levels at baseline or after cytokine stimulation did not significantly upregulate CD98 expression. These findings support a model in which peripheral blood NK cells mainly cover their energy demands through readily available glucose, while CD56^bright^ NK cells, and in particular that large population of tissue-located CD56^bright^ NK cells, obtain part of their energy demands via amino acids instead of glucose when activated.

Lastly, we examined the expression of CD71, a transporter for transferrin. CD71 has been described as a marker for T cell activation or proliferation [40]. Additionally, loss of *TFRC*, the gene encoding for CD71, has been demonstrated to cause a combined immunodeficiency in two families [41]. Prior to any stimulation, NK cells from tissues and peripheral blood had very similar expression levels of CD71, but post-cytokine stimulation, we observed a significantly higher expression of CD71 on peripheral blood and tissue-derived CD56^bright^ NK cells compared to CD56^dim^ NK cells from the same compartments. In liver samples and their paired blood samples, we observed that CXCR6^+^ CD56^dim^ NK cells in tissues had a higher expression of CD71 after stimulation compared to CXCR6^neg^ NK cells in tissues or CD56^dim^ NK cells from the peripheral blood. While the exact role of CD71 and iron in NK cell function remains unknown, parallels to T cell function suggest that iron might be required for expansion of NK cells, but also for their activation. We observed a higher expression of CD71 on CD56^bright^ NK cells than CD56^dim^ cells irrespective of the tissue compartment. As iron has been shown to be a critical cofactor in DNA synthesis [42] and as peripheral blood CD56^bright^ NK cells have been suggested to be less differentiated precursors of peripheral blood CD56^dim^ NK cells [5], the increased expression of CD71 on CD56^bright^ NK cells after stimulation might be a reflection of the proliferative capacity of these cells and the proliferative effect of IL-12 and IL-15 stimulation. Additionally, liver-resident NK cells might have to compete with hepatocytes, one of the main storage sites for iron in the human body apart from hemoglobin, and thus express higher levels of CD71. Taken together, our findings suggest that CD71 and iron metabolism might have an important role in NK cell function and tissue residency, which will merit further research in the future.

In conclusion, our data show that tissue-resident, tissue-derived and peripheral blood NK cells differ in their baseline expression of nutrient transporters and their ability to upregulate these transporters in response to low-dose cytokine stimulation. Limitations of this study are that samples were obtained from individuals that underwent liver transplantation, liver resection or spleen resection for medical indications, and it remains unclear how these findings translate into findings in healthy individuals. Furthermore, only changes in three nutrient transporters were assessed, and immune cells can express a large number of different nutrient receptors, including receptors involved in the transport of other nutrients such as lipids. The observed differences between peripheral blood NK cells and tissue-derived CXCR6^neg^ NK cells furthermore suggest that either a population of tissue-resident NK cells within this tissue-resident population is skewing our results or that tissues can affect cells passing through them in a more profound way than was previously anticipated. These data highlight the importance of studying immune cells in different tissue compartments, as the different nutritional conditions in these compartments might have critical effects on the immune cells that can be found in these tissues. Future studies should include more comprehensive assessments of the large number of nutrient transporters to obtain a more complete picture of the metabolic adaptations of immune cells in different compartments, both in health and disease.

## Supporting information

S1 Fig Expression of Glut1, CD98 and CD71 at baseline and after incubation without cytokines: Samples were compared using Wilcoxon matched-pairs signed rank tests and multiplicity was controlled for by FDR testing. Bars indicate the median, significance was defined as p≤0.05 (*).
A. Expression (Median fluorescence intensity, MdFI) of Glut1 on unincubated (“Fresh”) and incubated but unstimulated (“Rested”) CD56^bright^CD16^-^ (left) and CD56^dim^CD16^+^ (right) tissue-resident (TR), tissue-derived (TD) and peripheral blood (PB) NK cells from paired liver-blood (left diagram, n = 12) and spleen-blood (right diagram, n = 11) samples.
B. Expression (Median fluorescence intensity, MdFI) of CD98 on unincubated (“Fresh”) and incubated but unstimulated (“Rested”) CD56^bright^CD16^-^ (left) and CD56^dim^CD16^+^ (right) tissue-resident (TR), tissue-derived (TD) and peripheral blood (PB) NK cells from paired liver-blood (left diagram, n = 12) and spleen-blood (right diagram, n = 11) samples.
C. Expression (Median fluorescence intensity, MdFI) of CD71 on unincubated (“Fresh”) and incubated but unstimulated (“Rested”) CD56^bright^CD16^-^ (left) and CD56^dim^CD16^+^ (right) tissue-resident (TR), tissue-derived (TD) and peripheral blood (PB) NK cells from paired liver-blood (left diagram, n = 12) and spleen-blood (right diagram, n = 11) samples.
(TIFF)

S2 Fig Expression of Glut1, CD98 and CD71 at after incubation without cytokines and with cytokines: Samples were compared using Wilcoxon matched-pairs signed rank tests and multiplicity was controlled for by FDR testing. Bars indicate the median, significance was defined as p≤0.05 (*).
A. Expression (Median fluorescence intensity, MdFI) of Glut1 on unstimulated (“Rested”) and stimulated CD56^bright^CD16^-^ (left) and CD56^dim^CD16^+^ (right) tissue-resident (TR), tissue-derived (TD) and peripheral blood (PB) NK cells from paired liver-blood (left diagram, n = 12) and spleen-blood (right diagram, n = 11) samples.
B. Expression (Median fluorescence intensity, MdFI) of CD98 on unstimulated (“Rested”) and stimulated CD56^bright^CD16^-^ (left) and CD56^dim^CD16^+^ (right) tissue-resident (TR), tissue-derived (TD) and peripheral blood (PB) NK cells from paired liver-blood (left diagram, n = 12) and spleen-blood (right diagram, n = 11) samples.
C. Expression (Median fluorescence intensity, MdFI) of CD71 on unstimulated (“Rested”) and stimulated CD56^bright^CD16^-^ (left) and CD56^dim^CD16^+^ (right) tissue-resident (TR), tissue-derived (TD) and peripheral blood (PB) NK cells from paired liver-blood (left diagram, n = 12) and spleen-blood (right diagram, n = 11) samples.
(TIFF)

S1 Table Median and interquartile range (IQR) of the median fluorescence intensity (MdFI) of Glut1, CD98 and CD71 expression on tissue-resident (TR), tissue-derived (TD) and peripheral blood (PB) NK cells from liver and spleen donors. (XLSX)

S2 Table Median and interquartile range (IQR) of %CD56bright NK cells, %CXCR6+ among CD56bright NK cells and %CXCR6+ among CD56dim NK cells in tissue and blood of liver and spleen tissue donors after overnight incubation without ("Rested") or with 5ng/mL of IL-12 and 2.5ng IL-15/ml. (XLSX)

S3 Table Median and interquartile range (IQR) of the median fluorescence intensity (MdFI) and fold difference of Glut1 expression on tissue-resident (TR), tissue-derived (TD) and peripheral blood (PB) NK cells incubated without ("rested") or with ("stimulated") cytokines from liver and spleen donors. (XLSX)

S4 Table Median and interquartile range (IQR) of the median fluorescence intensity (MdFI) and fold difference of CD98 expression on tissue-resident (TR), tissue-derived (TD) and peripheral blood (PB) NK cells incubated without ("rested") or with ("stimulated") cytokines from liver and spleen donors. (XLSX)

S5 Table Median and interquartile range (IQR) of the median fluorescence intensity (MdFI) and fold difference of CD71 expression on tissue-resident (TR), tissue-derived (TD) and peripheral blood (PB) NK cells incubated without ("rested") or with ("stimulated") cytokines from liver and spleen donors. (XLSX)
