# Supplementary figures and images for: Tissue-resident NK cells differ in their expression profile of the nutrient transporters Glut1, CD98 and CD71
Source: PLoS One. 2018 Jul 20;13(7):e0201170. doi: 10.1371/journal.pone.0201170 (PMC6054388; doi:10.1371/journal.pone.0201170)

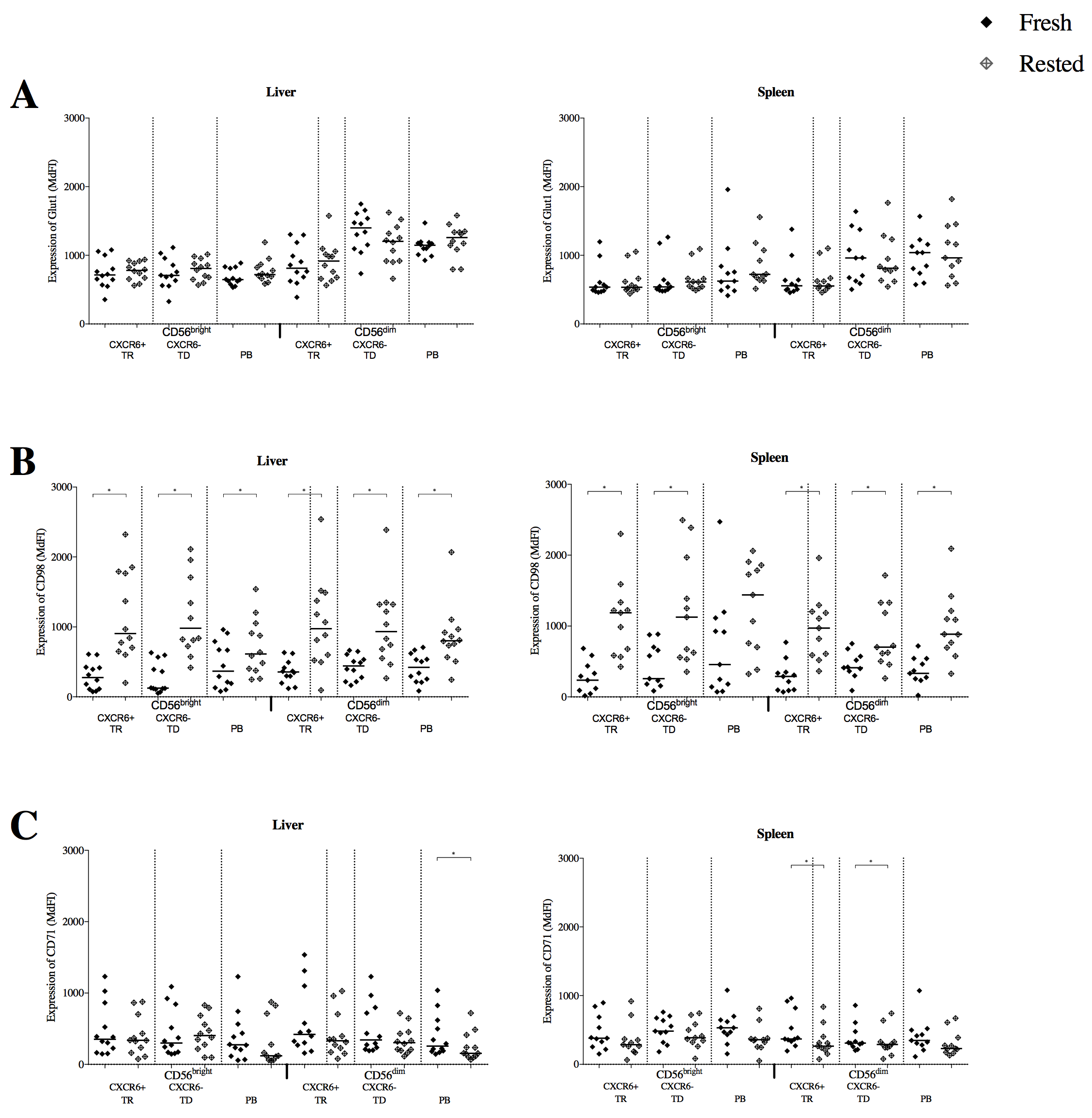

Supplement: S1 Fig — Bars indicate the median, significance was defined as p≤0.05 (*). A. Expression (Median fluorescence intensity, MdFI) of Glut1 on unincubated (“Fresh”) and incubated but unstimulated (“Rested”) CD56brightCD16- (left) and CD56dimCD16+ (right) tissue-resident (TR), tissue-derived (TD) and peripheral blood (PB) NK cells from paired liver-blood (left diagram, n = 12) and spleen-blood (right diagram, n = 11) samples. B. Expression (Median fluorescence intensity, MdFI) of CD98 on unincubated (“Fresh”) and incubated but unstimulated (“Rested”) CD56brightCD16- (left) and CD56dimCD16+ (right) tissue-resident (TR), tissue-derived (TD) and peripheral blood (PB) NK cells from paired liver-blood (left diagram, n = 12) and spleen-blood (right diagram, n = 11) samples. C. Expression (Median fluorescence intensity, MdFI) of CD71 on unincubated (“Fresh”) and incubated but unstimulated (“Rested”) CD56brightCD16- (left) and CD56dimCD16+ (right) tissue-resident (TR), tissue-derived (TD) and peripheral blood (PB) NK cells from paired liver-blood (left diagram, n = 12) and spleen-blood (right diagram, n = 11) samples. (TIFF) [file pone.0201170.s001.tiff]

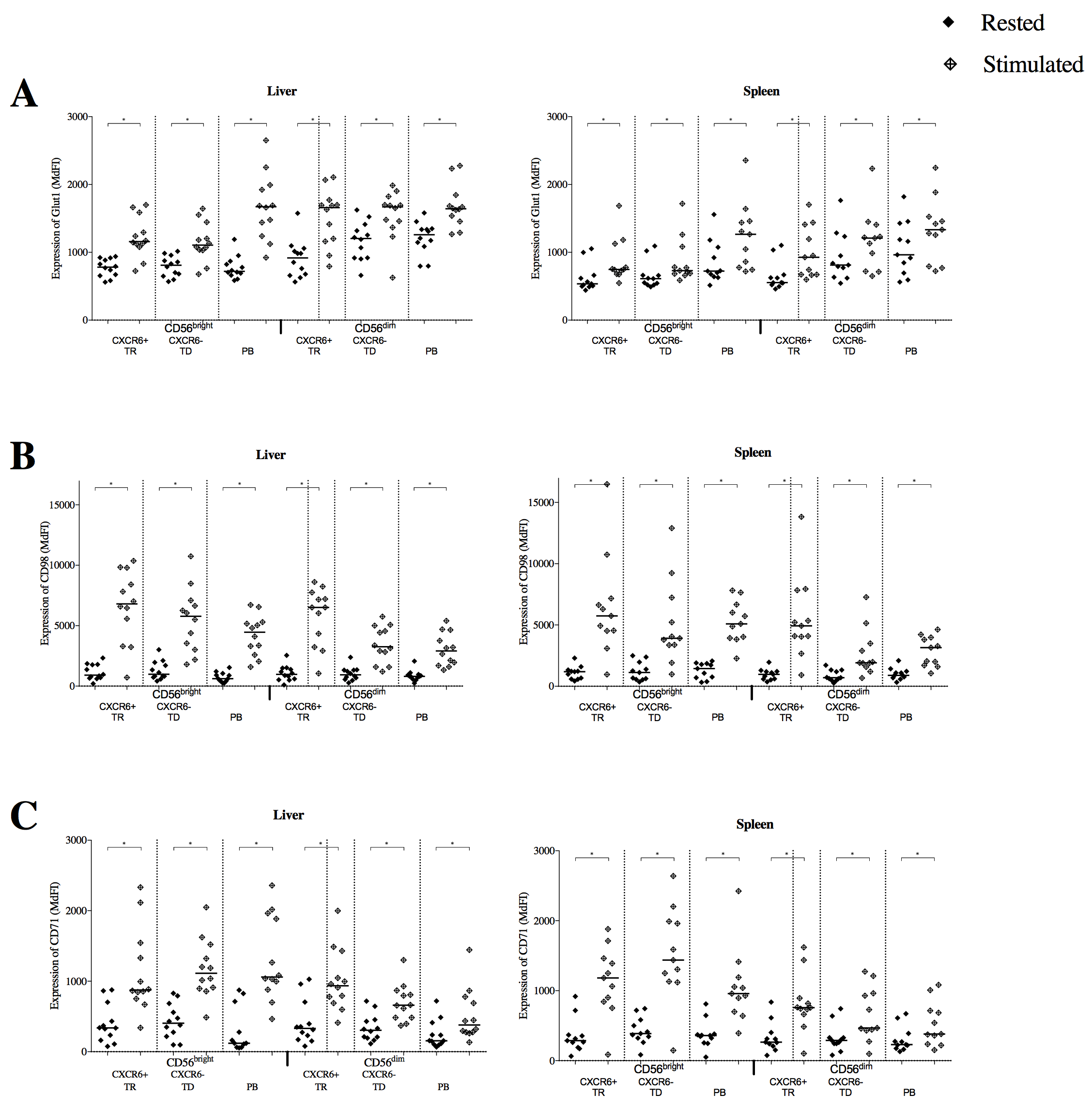

Supplement: S2 Fig — Bars indicate the median, significance was defined as p≤0.05 (*). A. Expression (Median fluorescence intensity, MdFI) of Glut1 on unstimulated (“Rested”) and stimulated CD56brightCD16- (left) and CD56dimCD16+ (right) tissue-resident (TR), tissue-derived (TD) and peripheral blood (PB) NK cells from paired liver-blood (left diagram, n = 12) and spleen-blood (right diagram, n = 11) samples. B. Expression (Median fluorescence intensity, MdFI) of CD98 on unstimulated (“Rested”) and stimulated CD56brightCD16- (left) and CD56dimCD16+ (right) tissue-resident (TR), tissue-derived (TD) and peripheral blood (PB) NK cells from paired liver-blood (left diagram, n = 12) and spleen-blood (right diagram, n = 11) samples. C. Expression (Median fluorescence intensity, MdFI) of CD71 on unstimulated (“Rested”) and stimulated CD56brightCD16- (left) and CD56dimCD16+ (right) tissue-resident (TR), tissue-derived (TD) and peripheral blood (PB) NK cells from paired liver-blood (left diagram, n = 12) and spleen-blood (right diagram, n = 11) samples. (TIFF) [file pone.0201170.s002.tiff]
